# Supplementary material for: Identification and functional analysis of non-coding regulatory small RNA FenSr3 in Bacillus amyloliquefaciens LPB-18
Source: PeerJ. 2023 May 15;11:e15236. doi: 10.7717/peerj.15236 (PMC10194069; doi:10.7717/peerj.15236)
Supplement: Supplemental Information 4 [file peerj-11-15236-s004.zip › KO/CK-vs-T1_map/map04341.html]

KEGG PATHWAY: Hedgehog signaling pathway - fly - Reference pathway


|  |  |
| --- | --- |
| **Hedgehog signaling pathway - fly - Reference pathway** |  |

[
Pathway menu
| Organism menu
| Pathway entry
| Show description
| User data mapping
]

Hedgehog (Hh) signaling plays essential roles in embryonic development and adult tissue homeostasis. It is first elucidated by genetic studies in Drosophila and the core of the Hh signaling pathway is conserved in evolution. The Hh signaling components include an important complex composed of Cos2, Fu, SuFu and Ci. In the absence of Hh, Ptc inhibits Smo and the complex promotes the formation of the Ci's repressor form CiR. In the presentation of Hh, binding Hh to Ptc relieves Smo repression, which results in Ci's activation and then the increased transcription of Hh target genes in the nucleus.

|  |  |  |
| --- | --- | --- |
| Reference pathway | 184% 150% 122% 100% 82% 67% 55% | 图片下载 |
